# Endoglin mediates the tumor‐ and metastasis‐promoting traits of stromal myofibroblasts in human breast carcinomas

**DOI:** 10.1002/1878-0261.70074

**Published:** 2025-07-11

**Authors:** Shoki Okubo, Yoshihiro Mezawa, Zixu Wang, Ahmet Acar, Yasuhiko Ito, Atsushi Takano, Yohei Miyagi, Tomoyuki Yokose, Toshinari Yamashita, Yataro Daigo, Takuya Shirakihara, Akira Orimo

**Affiliations:** ^1^ Department of Gastroenterology, Graduate School of Medicine Juntendo University Tokyo Japan; ^2^ Department of Molecular Pathogenesis, Graduate School of Medicine Juntendo University Tokyo Japan; ^3^ Department of Pathology and Oncology, Graduate School of Medicine Juntendo University Tokyo Japan; ^4^ Department of Biological Sciences Middle East Technical University Ankara Turkey; ^5^ Department of Immunological Diagnosis, Graduate School of Medicine Juntendo University Tokyo Japan; ^6^ Center for Antibody and Vaccine Therapy, Institute of Medical Science, Research Hospital The University of Tokyo Japan; ^7^ Department of Medical Oncology and Cancer Center Shiga University of Medical Science Otsu Japan; ^8^ Center for Advanced Medicine against Cancer Shiga University of Medical Science Otsu Japan; ^9^ Molecular Pathology and Genetics Division Kanagawa Cancer Center Research Institute Yokohama Japan; ^10^ Department of Pathology Kanagawa Cancer Center Yokohama Japan; ^11^ Department of Breast and Endocrine Surgery Kanagawa Cancer Center Yokohama Japan

**Keywords:** breast cancer, endoglin, myofibroblastic carcinoma‐associated fibroblasts, TGF‐β1, TGF‐β‐Smad2/3 signaling, tumor microenvironment

## Abstract

Carcinoma‐associated fibroblasts (CAFs), which are abundant in the tumor microenvironment, influence cancer hallmarks. We previously described transforming growth factor‐β (TGF‐β)–Smad2/3 signaling as being activated in myofibroblastic CAFs (myCAFs) in an autocrine fashion by increasing TGF‐β production. However, factors regulating such autocrine TGF‐β signaling remain poorly understood. Herein, we show that the abundance of endoglin (ENG), a TGF‐β superfamily coreceptor expressed on human breast myCAFs, is significantly associated with poorer outcomes of breast cancer patients. Inhibition of *ENG* expression on myCAFs not only suppressed the TGF‐β–Smad2/3 pathway and TGF‐β1 expression but also attenuated the ability of myCAF to promote primary tumor growth and metastasis. Mechanistically, ENG facilitates TGF‐β–Smad2/3 signaling in myCAFs, presumably through association with a TGF‐β ligand–receptor complex, leading to self‐stimulating TGF‐β1 production. Stromal TGF‐β1, in turn, induces partial epithelial–mesenchymal transition in cancer cells in a paracrine manner, resulting in suppression of primary tumor growth and promotion of invasion and metastasis. ENG‐primed TGF‐β autocrine signaling also produces other factors that could mediate primary tumor growth promotion by myCAFs. Collectively, these findings suggest that ENG‐primed TGF‐β autocrine and paracrine signaling mediates tumor‐ and metastasis‐promoting abilities of myCAFs.

## Graphical abstract

Carcinoma‐associated fibroblasts (CAFs) in tumors influence cancer progression. We identified endoglin (ENG) as a key factor in TGF‐β signaling in myofibroblastic CAFs (myCAFs), linked to poor breast cancer outcomes. Inhibiting ENG on myCAFs suppressed the TGF‐β‐Smad2/3 pathway, reducing primary tumor growth and metastasis. ENG facilitates autocrine TGF‐β1 production in myCAFs and promotes tumor progression through the induction of partial epithelial‐mesenchymal transition in cancer cells in a paracrine fashion.

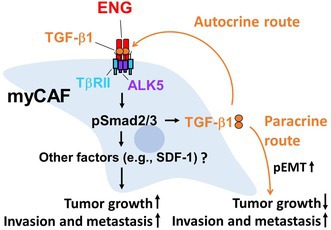

**Abbreviations**
ALK: TGF‐β type I/activin receptor‐like kinase
BMPs: bone morphogenetic proteins
CAFs: carcinoma‐associated fibroblasts
cPARP: cleaved poly‐ADP‐ribose polymerase
DCIS: ductal carcinoma *in situ*
ECM: extracellular matrix
ECs: endothelial cells
ENG: endoglin
exp‐CAFs: experimentally generated CAFs
FFPE: formalin‐fixed paraffin‐embedded
HE: hematoxylin and eosin
IL‐11: interleukin‐11
MPO: myeloperoxidase
myCAFs: myofibroblastic CAFs
PDAC: pancreatic ductal adenocarcinoma
pEMT: partial epithelial–mesenchymal transition
POSTN: periostin
SDF‐1: stromal cell‐derived factor‐1
TGF‐β: transforming growth factor‐β
α‐SMA: α‐smooth muscle actin

## 1 Introduction

The tumor microenvironment embedded in the extracellular matrix (ECM) consists of stromal cells, such as fibroblasts, inflammatory cells, immune cells, and vascular endothelial cells (ECs), which interact with carcinoma cells to affect tumorigenesis [1, 2, 3]. Carcinoma‐associated fibroblasts (CAFs), predominantly present in the tumor‐associated stroma, are composed of distinct fibroblast subpopulations in either a tumor‐promoting or a tumor‐suppressing state [4, 5, 6, 7, 8]. The generation of such distinct fibroblast subpopulations is regulated under the influence of various cells of origin, stimuli from the tumor microenvironment, and phenotypic plasticity varying among activated fibroblast states during tumor progression [4]. CAFs as a whole influence the hallmarks of tumors, promoting tumor growth, invasion, metastasis, neoangiogenesis, inflammation, immunosuppression, and drug resistance [9, 10, 11].

Transforming growth factor‐β (TGF‐β) induces α‐smooth muscle actin (α‐SMA) expression, indicative of the myofibroblastic state, and stimulates ECM production in fibroblasts, generating a desmoplastic stromal reaction during wound healing, fibrosis, and tumorigenesis [12, 13, 14]. A large number of TGF‐β‐induced myofibroblastic CAFs (myCAFs) are also often observed in various human cancers, including those of the breast [15].

Activation of TGF‐β signaling in stromal fibroblasts correlates with poorer outcomes in patients suffering from various carcinomas [16, 17, 18]. Previous studies have demonstrated that activated stromal TGF‐β signaling encourages CAFs to produce tumor‐promoting cytokines, such as interleukin‐11 (IL‐11), thereby promoting metastasis [18], and also to trigger TGF‐β production, which in turn stimulates TGF‐β‐Smad2/3 signaling in an autocrine fashion and remodeling of the ECM [19]. Paracrine stromal TGF‐β induces partial epithelial–mesenchymal transition (pEMT) in apposed tumor cells to suppress tumor cell proliferation and growth [20] and to stimulate invasion and metastasis [21]. Collectively, these observations demonstrate that CAFs with TGF‐β signal activation are capable of promoting tumor progression via the production of different paracrine factors involving TGF‐β.

Previously, we described CAFs as progressively increasing TGF‐β‐Smad2/3 signaling in an autocrine fashion during tumor progression, resulting in the acquisition of myofibroblastic abilities [19]. However, how such TGF‐β autocrine signaling becomes established in myCAFs during tumor progression remains unclear. Thus, we reasoned that endoglin (ENG)/CD105, which interacts with receptor complexes of the TGF‐β superfamily [12, 22], may regulate TGF‐β signaling activation and tumor‐promoting traits in myCAFs.

ENG is a 180 kDa, type‐I transmembrane glycoprotein composed of disulfide‐linked subunits of 95 kDa, which is highly expressed on vascular ECs at sites of active angiogenesis [23, 24]. ENG expression is upregulated in myofibroblasts present in tumors, injured tissues, and sites of fibrosis [25, 26, 27, 28]. ENG expression is also required not only for proliferation and viability in prostate‐derived stromal cells [29] but also for the myogenic differentiation from neural crest stem cells [30]. ENG‐positive or ‐negative pancreatic fibroblast populations are present in normal and cancerous regions of the pancreas. The former is tumor‐prone, while the latter is tumor‐suppressive due to its role in antitumor immunity [31]. ENG‐positive staining in CAF‐rich stroma is reportedly associated with poorer clinical outcomes in breast and colorectal cancer patients [25, 32] and resistance to androgen deprivation therapy in prostate cancer patients [33]. Increased *ENG* mRNA expression was also detected by RNA‐sequencing in human breast CAFs, which were generated in our previous study [34]. However, whether ENG contributes to TGF‐β signaling activation and tumor progression in human breast myCAFs has yet to be addressed. Thus, we aimed to elucidate the roles of ENG expression in regulating TGF‐β signaling and tumor‐ and metastasis‐promoting traits in myCAFs.

## 2 Materials and methods

### 2.1 Plasmid construction

Human *ENG* construct [35] was kindly offered by Calvin P.H. Vary (Center for Molecular Medicine, Maine Medical Center Research Institute, Scarborough, Maine) and cloned into the pWZLblast retroviral vector. A constitutively active form of the swine *TGF‐β1 (TGFB1)* vector, pPK9a [36], was generously provided by Lalage M. Wakefield (National Cancer Institute, Bethesda, MD, USA) and cloned into the pBabe‐neo retroviral vector and the pENTR1A no ccDB (w48‐1; Addgene #17398) vector [37]. *ECFP* was subcloned into pENTR1A no ccDB (w48‐1) from Histac pcDNA3.1 (RDB12840) [38]. CS‐UbC‐RfA‐IRES2‐hKO1 (RDB08350) [39] and each of the pENTR vectors were incubated with LR clonase (Invitrogen, Carlsbad, CA, USA) for generating CS‐UbC‐swine TGFB1‐IRES2‐hKO1 and CS‐UbC‐ECFP‐IRES2‐hKO1 according to the manufacturer's manual. The shRNA oligonucleotides against *GFP*, *ENG*, and *SMAD4* genes were generated and cloned into lentivirus‐derived pLKO‐hygro‐vectors [40]. Target sequences are listed in Table S1.

### 2.2 Cell lines

Cell lines of human mammary fibroblasts and experimentally generated CAFs (exp‐CAFs) used in this study were established in our previous studies [19]. Briefly, a breast tissue specimen was dissected from a healthy donor who had undergone a reduction mammoplasty procedure. Stromal fibroblasts were primary cultured in DMEM high‐glucose GlutaMAX supplemented with 10% calf serum (Gibco, North Andover, MA, USA), as described previously [41]. For immortalization, the retroviral pMIG (MSCVIRES‐GFP) vector, expressing *hTERT* and *GFP*, and the primary cultured human mammary fibroblasts were sequentially infected with a pBabe‐puro vector encoding a puromycin resistance gene as described previously [19]. To generate exp‐CAFs, the resulting immortalized GFP‐positive puromycin‐resistant human mammary fibroblasts were comingled with MCF‐7‐ras tumor cells prior to subcutaneous injection into immunodeficient nude mice. The advanced tumors were resected on various days after injection and selected by puromycin (1 μg·mL^−1^) treatment in culture to isolate the injected puromycin‐resistant human mammary fibroblasts, as previously described [19]. The control fibroblasts were also extracted from the recipient nude mice, in which immortalized GFP‐positive puromycin‐resistant human mammary fibroblasts had been injected without any carcinoma cells, followed by the puromycin treatment (1 μg·mL^−1^) for selection and propagation in culture. MCF10DCIS.com cells (referred to as DCIS cells) [42] (Asterand Bioscience, Detroit, MI, USA) were cultured in DMEM/F12 with GlutaMAX (Gibco) supplemented with 5% fetal bovine serum (FBS) and penicillin–streptomycin.

### 2.3 Tumor xenograft assay

Male highly immunodeficient NOD/Shi‐scid IL2R γ null (NOG) mice at 6 weeks of age were purchased from the Central Institute for Experimental Animals (Kanagawa, Japan). The mice were kept under germ‐free and specific pathogen‐free conditions, and the experiments were approved by the Animal Research Ethics Committee of the Juntendo Faculty of Medicine (approval number: 1035). 1 × 10^5^ DCIS cells and 3 × 10^5^ exp‐CAFs or control fibroblasts expressing various shRNAs, ECFP, or active TGF‐β1 were suspended in 200 μL of culture medium with 50% Matrigel (BD Biosciences, Franklin Lakes, NJ, USA) prior to subcutaneous injections into the right and left flanks of eight‐week‐old NOG mice using a 25‐gauge needle, as previously described [21]. The primary tumors were then resected at 21–25 days after injection and weighed, followed by measurement of metastatic nodules using a ruler in the lungs dissected from mice at 65 days after injection. Tumors formed by DCIS cells admixed with exp‐CAF2‐shENG‐2 cells expressing ECFP or active TGF‐β1 were removed at 30 days after injection, and metastatic nodules in the lungs were measured 50–53 days after injection.

The tumor volume of nodules was calculated with the formula 4/3πr^3^ and the metastatic index was calculated as metastatic nodule volume (mm^3^)/primary tumor weight (g). As a guideline for humanitarian endpoints, mice were euthanized when the tumor weight exceeded 10% of the mouse's body weight or the tumor diameter exceeded 20 mm. This humane endpoint is based on “Guidelines for the Proper Conduct of Animal Experiments” created by the Science Council of Japan in 2006.

### 2.4 Virus infections

Retroviral and lentivirus infections were performed as described previously [43]. Briefly, a pLKO.1‐shRNA‐hygro, CS‐UbC‐swine TGFB1‐IRES2‐hKO1, or CS‐UbC‐ECFP‐IRES2‐hKO1 vector was cotransfected with lentiviral envelope and packaging plasmids including pCMV‐dR8.2dvpr and pCMV‐VSVG into HEK293T cells using Fugene6 (Promega, Madison, WI, USA). A pBabe‐neo vector encoding *ENG* or active swine *TGFB1* cDNA was also cotransfected with retroviral envelope and packaging plasmids including pUMVC3‐gag‐pol and pCMV‐VSVG into HEK293T cells. Conditioned media containing viruses were applied onto fibroblasts for infection prior to selection with hygromycin (50 μg·mL^−1^) for 5–7 days. Humanized kusabira‐orange 1 (hKO1) expressing exp‐CAF2 cells were sorted using MoFlo Astrios (BECKMAN COULTER, Brea, CA, USA) after introduction with CS‐UbC‐swine TGFB1 or ‐ECFP‐IRES2‐hKO1. These cells were then expanded in culture prior to the introduction of a pLKO.1‐shENG‐2‐hygro vector.

### 2.5 Western blot analysis

5 × 10^5^ fibroblasts were seeded onto a 6‐cm dish and cultured for 24 h in DMEM high‐glucose GlutaMAX with 2% FBS. Whole cells were lysed employing SDS gel loading buffer. The lysed protein was separated by SDS/polyacrylamide gel electrophoresis on an 8–12% acrylamide gel and transferred to a PVDF membrane. 5% skim milk was used for blocking. Then, primary antibodies were incubated in TBS‐T (0.05 m Tris pH 7.6, 0.15 m NaCl and 0.05% Tween 20) with 10% FBS. Detection was performed using a ChemiDoc MP imaging system (BIO RAD, Hercules, CA, USA). Quantification of the band intensity was performed using the Image J software.

### 2.6 Real‐time PCR analysis

Total RNA was extracted using NucleoSpin RNAII (Macherey‐Nagel, Düren, Germany), followed by cDNA synthesis using SuperScript III reverse transcriptase (Invitrogen) in accordance with the manufacturer's protocol. 7500 Fast Real‐Time PCR systems (Applied Biosystems, Waltham, MA, USA) were used for real‐time PCR analysis. Results were evaluated with the 7500 software (Applied Biosystems). Data for each sample were normalized relative to the expression levels of *β2‐microglobulin (B2M)* and *GAPDH* genes. Primers used for PCR analyses are described in Table S1.

### 2.7 Flow cytometry

1–5 × 10^5^ human mammary fibroblasts were stained with antihuman ENG mouse antibody conjugated with Alexa 647 in 1% FBS‐PBS, followed by 4',6‐diamidino‐2‐phenylindole (DAPI) staining to eliminate nonviable cells. Those cells were analyzed using a BD LSRFortessa cell analyzer (BD Biosciences). The antibodies used to detect ENG are listed in Table S1.

### 2.8 Evaluation of the number of tumor budding

The tumor budding count was performed on tumor sections stained by hematoxylin and eosin (HE). Tumor budding was defined as the rounded tumor cluster containing 4–100 tumor cells within stroma. Five fields (×200 magnification per field) on a tumor section were evaluated in each group (*n* = 12).

### 2.9 Evaluation of angiogenesis

To evaluate microvascular density, sections prepared from tumor xenografts in each group (*n* = 8–13) were immunostained using anti‐CD31 antibody. The number of CD31‐positive capillary vessels was quantified in a total of 10 fields (×400 magnification per field) per section, as reported previously [41]. To assess the vascular lumen volume, a 315‐point graticule was also employed over the same tissue section and the number of graticular points that fell within the lumen of vessels was counted.

### 2.10 Double immunostaining of human breast tissues

This study was conducted in accordance with the Declaration of Helsinki and was approved by the Institutional Review Board of Juntendo University (approval number: H16‐0160). In accordance with national ethical guidelines, written informed consent was not required for this retrospective study using de‐identified samples obtained from formalin‐fixed specimens that had been prepared for pathological diagnosis following surgery. Instead, an opt‐out approach was employed. Information about the study was publicly disclosed on the institution's website, and patients were given the opportunity to decline participation. The specimens were collected at Juntendo University between January 2012 and December 2014. Immunostaining was performed as previously described, with some modifications to perform double immunostaining [44]. 3‐μm‐thick sections were prepared and deparaffinized. The slides were then treated with 0.3% H_2_O_2_ in methanol for 20 min at room temperature. Antigen retrieval was performed by autoclaving in citrate buffer at pH 6.0 for 20 min at 121 °C. The slides were incubated with anti‐ENG antibody at 4 °C overnight, followed by incubation with the secondary antibody for 1 h at room temperature and treatment with 3,3'diaminobenzidine as the chromogen. After antigen retrieval in citrate buffer at pH 6.0 for 20 min at 121 °C, the slides were subsequently incubated with antiperiostin (POSTN), −α‐SMA, or ‐CD31 antibodies at 4 °C overnight. They were then incubated with the secondary antibody for 1 h at room temperature and treated with Vulcan Fast Red as the chromogen and hematoxylin for nuclear counterstaining. We counted POSTN‐, α‐SMA‐, and CD31‐positive cells within ENG‐positive fibroblast‐like cells in a total of 10 fields (×400 magnification per field) per section to evaluate the double staining.

### 2.11 Tissue microarray

Tissue microarrays were constructed, immunostained, and analyzed using 232 FFPE human primary breast cancer specimens, as reported previously [44, 45]. Specimens were surgically resected at the Kanagawa Cancer Center prior to the generation of FFPE sections. Individual institutional ethics committees approved the use of all clinical materials in this study. Experiments were performed in accordance with all guidelines and regulations indicated by these committees.

The tissue area for sampling was selected based on visual alignment with the corresponding HE‐stained section on a slide. Several tissue cores (diameter 0.6 mm; height 3–4 mm) taken from a donor tumor block were placed in a recipient paraffin block using a tissue microarrayer (Beecher Instruments, Sun Prairie, WI, USA). The resulting microarray blocks were used for immunohistochemical analysis. The sections were stained using anti‐ENG antibody according to the conditions described earlier. Immunohistochemical scores for ENG staining on stromal fibroblast‐like cells were determined by a researcher with no prior knowledge of the clinicopathological results, as follows: positive (≧ 10% of total area) and negative (< 10% of total area) for stromal ENG staining in tumor‐associated stroma, as described previously [43, 44].

### 2.12 Statistical analysis

Statistical analyses were performed using Student's *t*‐test or the Mann–Whitney *U* test. Values of *P* < 0.05 were considered to indicate a statistically significant difference.

### 2.13 Chemicals and antibodies

SB431542 was purchased from Sigma‐Aldrich (Milwaukee, WI, USA) and the primary antibodies used are listed in Table S1.

## 3 Results

### 3.1 Stronger ENG staining on myCAFs is significantly associated with poorer outcomes in breast cancer patients

Since our previous work indicated that TGF‐β signaling becomes stably activated in myCAFs during tumor progression [19], we speculated that the dysregulated expression of ENG, a TGF‐β superfamily coreceptor, may be involved in TGF‐β signaling activation in these fibroblasts.

**Fig. 1:**
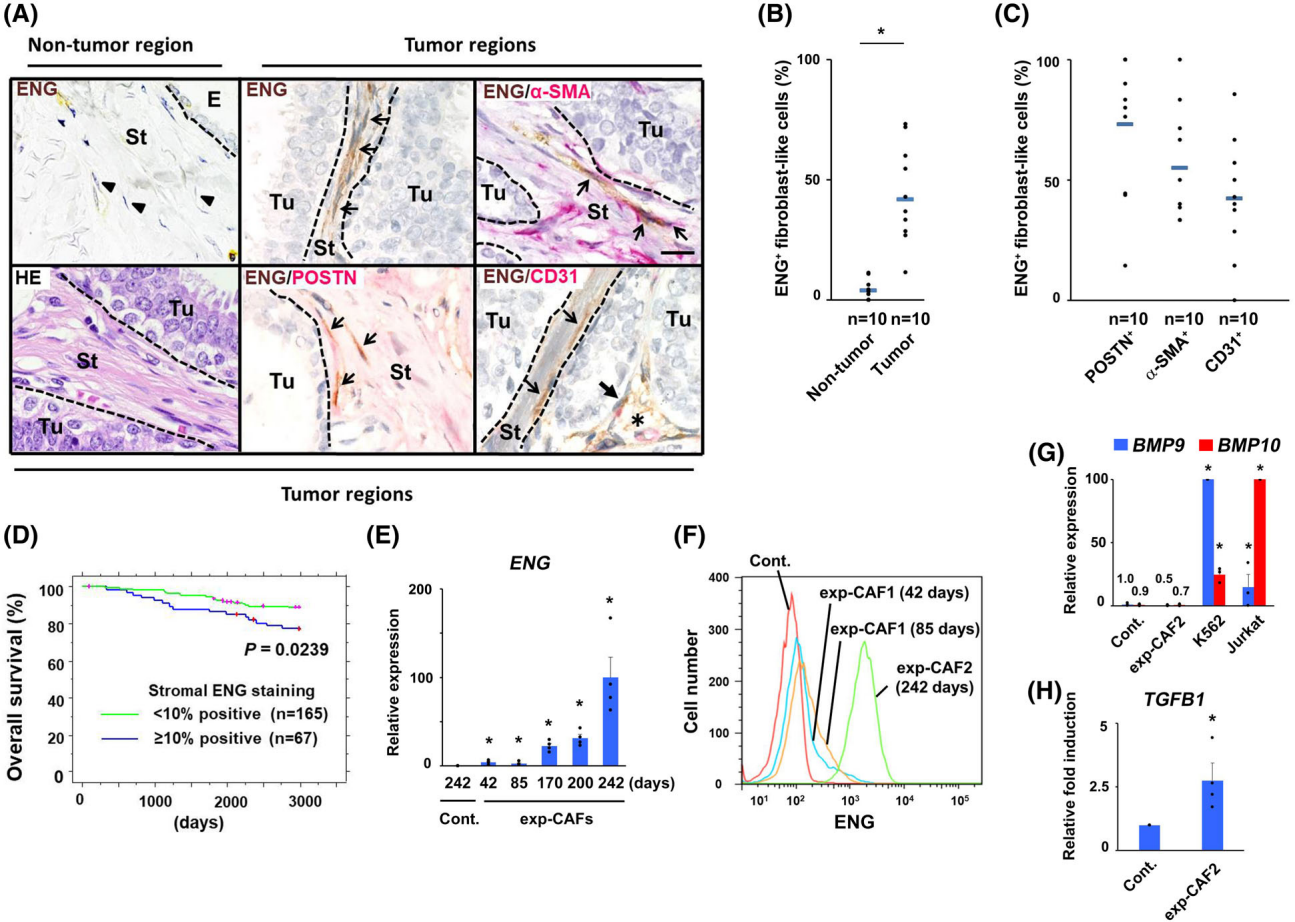
Stronger ENG staining in myCAFs is associated with poorer outcomes in human breast cancer patients. (A) Immunohistochemistry of tumor sections prepared from a breast cancer patient (an estrogen receptor‐positive type) using antibodies for endoglin (ENG), periostin (POSTN), α‐SMA, and CD31. More ENG^+^ fibroblast‐like cells (simple arrow, upper middle) are shown in tumor‐associated stroma, as compared to those (arrowhead, upper left) in the interlobular area of non‐tumor regions. ENG^+^POSTN^+^ (simple arrow, lower middle) and ENG^+^α‐SMA^+^ (simple arrow, upper right) myofibroblastic carcinoma‐associated fibroblasts (myCAFs) are indicated in tumor regions. ENG^+^CD31^−^ fibroblast‐like cells (simple arrow, lower right) and ENG^+^CD31^+^ vascular endothelial cells (ECs) (triangular arrow, lower right) associated with a capillary vessel (asterisk) are shown. Areas corresponding to tumor epithelium, normal epithelium, and stroma are divided by a dashed line. E: normal epithelium Tu: tumor epithelium St: stroma Scale bar, 20 μm. (B) A larger proportion (%) of ENG‐positive fibroblast‐like cells in tumor regions relative to non‐tumor regions. The proportion (%) of ENG‐positive fibroblast‐like cells in tumor and non‐tumor lesions was calculated by quantification at ×400 magnification, 10 fields per section. Blue horizontal line means the average of all data points. A significant difference (*P* < 0.05) is indicated by an asterisk. (C) Among ENG^+^ fibroblast‐like cells, POSTN‐, α‐SMA‐, and CD31‐positive cell proportions were also evaluated in tumor regions by quantification at ×400 magnification, 10 fields per section. Blue horizontal line means the average of all data points. (D) Kaplan–Meier plot indicating overall survival of 232 breast cancer patients. Patients are classified based on the indicated stromal ENG staining (negative, < 10% positivity; strong/weak, ≥ 10% positivity) in human breast carcinomas. (E) Real‐time PCR analysis of control human mammary fibroblasts (Cont.) and experimentally generated CAFs (exp‐CAFs) using *ENG* gene‐specific primers. Relative expressions were calculated with exp‐CAF2 cells (exp‐CAFs at 242 days) taken as 100. Asterisks indicate a significant difference (*P* < 0.05) compared to Cont.; *n* = 4, each performed in triplicate. (F) Flow cytometry of the indicated cells using an anti‐ENG antibody. (G, H) Real‐time PCR analysis of the indicated cells using primers for the described genes. A significant difference (*P* < 0.05) as compared to control fibroblasts is indicated by an asterisk (*). The number of relative expression levels is indicated in cont. and exp‐CAF2 cells. *n* = 10 (B), *n* = 10 (C), *n* = 4 (F), *n* = 3 (G) and *n* = 4 (H) E, G, and H are performed in triplicate. Mann–Whitney *U* test (B), Student's *t*‐test (E, G, H), log‐rank test (D); error bars, SE (E, G, H).

To address this possibility, tumor sections were prepared from breast cancer patients for immunohistochemistry using antibodies for ENG and myofibroblast markers, including POSTN and α‐SMA. We observed a greater proportion of ENG‐positive fibroblast‐like cells with stronger ENG staining in tumor‐associated stroma, as compared with the stroma of noncancerous regions of the same specimen (Fig. 1A,B). These ENG^+^ fibroblast‐like cells were also positive for POSTN and α‐SMA in tumor stroma (Fig. 1A‐simple arrow). The proportion of POSTN‐ and α‐SMA‐positive ENG^+^ fibroblast‐like cells was also quantified to be 73% and 55%, respectively (Fig. 1C), indicating that ENG is present on myCAFs. ENG staining was negative on cancer cells, granulocytes, and macrophages (Fig. S1A).

Since ENG is also a well‐known marker for vascular ECs [46], the tumor sections were stained with anti‐CD31 (a marker for vascular ECs) and anti‐ENG antibodies. As anticipated, ENG^+^ vascular ECs histologically associated with vasculature stained positive for CD31 (ENG^+^CD31^+^ ECs, Fig. 1A‐triangle‐headed arrow). In contrast, spindle‐shaped ENG^+^ fibroblast‐like cells, which were not associated with vasculature, were negative for CD31 (ENG^+^CD31^−^ fibroblast‐like cells, Fig. 1A‐simple arrow) in tumor‐associated stroma. However, vascular ECs often appeared not to be associated with vasculature due to routine thin sectioning preparation, resulting in their morphological resemblance to fibroblasts. To determine how many ENG^+^ fibroblast‐like cells involve vascular ECs, a proportion of ENG^+^ fibroblast‐like cells positive for CD31 was quantified. 42% of the ENG^+^ fibroblast‐like cells were indeed positive for CD31 (Fig. 1C), suggesting that less than half of the ENG^+^ fibroblast‐like cell proportion represents ECs. Collectively, these data demonstrate that ENG is expressed by myCAFs and vascular ECs in the tumor stroma.

We observed intense ENG staining on stromal fibroblast‐like cells on tumor sections from two (estrogen receptor‐positive) of the 33 breast cancer patients (Fig. 1A and Fig. S1B). A previous report demonstrated higher proportions of ENG^+^ fibroblasts in intralobular areas relative to interlobular areas of normal human breast tissue [47], the ENG^+^ fibroblasts potentially resembling CAFs. Thus, we assessed ENG staining in noncancerous stromal regions in the above two patients, in whom ENG^+^ CAFs were clearly detected in the tumor regions. We observed an absence of ENG staining in both intralobular and interlobular areas of the noncancerous breast tissue (Fig. 1A and Fig. S1B), indicating that increased ENG staining is mainly on CAFs in human breast cancer samples of our cohort.

To more precisely evaluate ENG staining on stromal fibroblasts and better determine the clinical significance, we employed a larger cohort including 232 breast cancer patients (Table S2) and performed immunohistochemistry with an anti‐ENG antibody (Fig. S1C). Strongly and weakly positive (over 10%) stromal ENG staining, detected in 67 of the 232 tumors, was significantly associated with poorer outcomes of breast cancer patients as compared to those with negative (below 10%) stromal ENG staining by Kaplan–Meier analysis (Fig. 1D). A trend of poorer outcomes associated with the positive stromal ENG staining was also observed in multivariable Cox regression analysis (Table S3). A regression model showed weakly positive (over 10%) stromal ENG staining, detected in 47 of the 232 tumors, to be significantly associated with poorer outcomes (Table S4). However, strongly positive stromal ENG staining in 20 tumors was not significantly associated with poorer outcomes of breast cancer patients (Fig. S1D and Table S4), presumably due to a small number of patients. A previous report has consistently indicated high ENG staining on CD34‐negative stromal fibroblast‐like cells to be associated with poorer outcomes in 56 breast cancer patients [32]. We found no significant correlations of stromal ENG staining with pathological parameters, such as grading, pT factor, pN factor, estrogen receptor status, or human epithelial growth factor receptor‐2 status (Table S2).

### 3.2 ENG expression is progressively upregulated in human breast CAFs during tumor progression

Given the increased ENG staining on stromal myofibroblasts in human breast carcinomas, we investigated whether ENG expression is also upregulated in cultured CAFs. To address this possibility, we employed exp‐CAFs, which were previously shown to have myofibroblastic and tumor‐promoting abilities [19]. The immortalized human mammary fibroblasts were co‐injected with human breast carcinoma cells into recipient mice for periods of 42–242 days, prior to extraction of the injected human fibroblasts from the developing tumor xenografts to isolate exp‐CAFs in culture [19].

We found *ENG* mRNA expression to be upregulated progressively in exp‐CAFs extracted from 42‐, 85‐, 170‐, 200‐, and 242‐day‐old tumors as compared to that in control fibroblasts (Fig. 1E). The highest level of *ENG* expression was observed in exp‐CAFs extracted from 242‐day‐old tumors, designated exp‐CAF2 cells. In sharp contrast, mRNA expression of *betaglycan/TGF‐β type III receptor (TGFBR3)*, another coreceptor of TGF‐β, was decreased in exp‐CAF2 cells relative to control fibroblasts (Fig. S1E). Flow cytometry detecting the cell‐surface ENG protein also showed an increasing proportion of upregulated ENG‐positive cells in exp‐CAFs during tumor progression (Fig. 1F).

ENG is a coreceptor for bone morphogenetic proteins (BMPs) and TGF‐βs associating with TGF‐β type I/activin receptor‐like kinase (ALK) and type II receptors. ENG and ALK1 bind with high affinity to BMP9 and BMP10 on most ECs, while ENG and ALK5 bind to TGF‐βs on other cells, including myoblasts [48, 49, 50, 51]. Therefore, we sought to determine whether CAF‐produced BMP or TGF‐β associates with ENG. We observed *BMP9* and *BMP10* mRNA, highly expressed in K562 and Jurkat leukemia cells, to be undetectable in both control fibroblasts and exp‐CAF2 cells (Fig. 1G). In contrast, mRNA expression of *TGFB1*, which binds to ENG with high affinity [52], was increased in exp‐CAF2 cells by 2.8‐fold, as compared to that in control fibroblasts (Fig. 1H). This datum is consistent with our previous findings indicating substantially upregulated TGF‐β1 expression in exp‐CAF2 cells [19]. Taken together, these findings suggest that ENG is more likely associated with TGF‐β1 rather than BMP9 and BMP10 on human breast CAFs.

### 3.3 ENG facilitates TGF‐β‐Smad2/3 signaling in myCAFs, presumably via association with a TGF‐β ligand–receptor complex

**Fig. 2:**
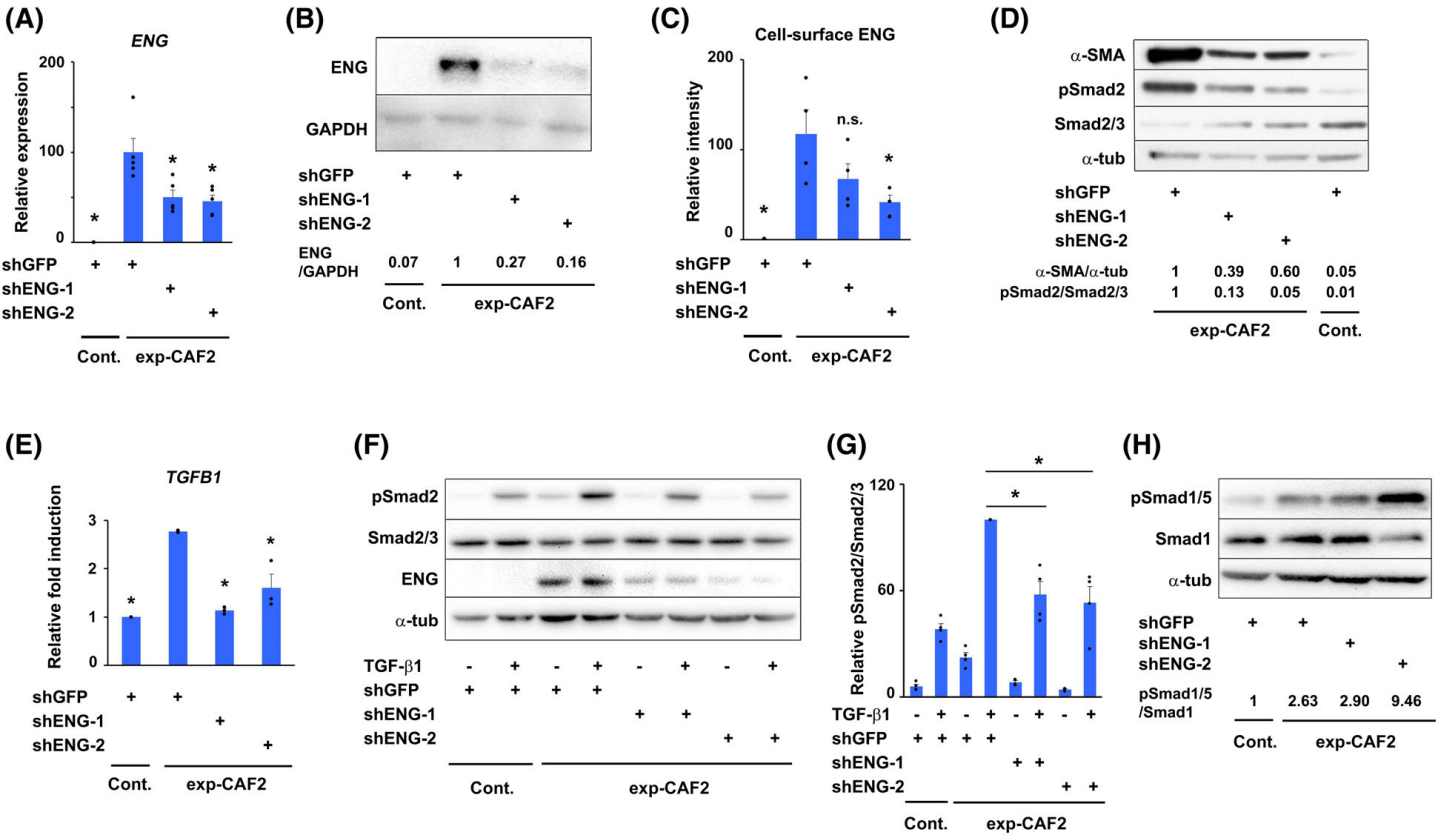
ENG facilitates TGF‐β‐Smad2/3 signaling in myCAFs, presumably through association with a TGF‐β ligand–receptor complex. (A) Real‐time PCR analysis of control fibroblasts (Cont.) and experimentally generated CAF2 (exp‐CAF2) cells expressing the indicated shRNA using *endoglin* (*ENG)* gene‐specific primers. A significant difference (*P* < 0.05) compared to exp‐CAF2‐shGFP cells is indicated by an asterisk; *n* = 5, each performed in triplicate. (B) Immunoblotting of the indicated cells expressing the described shRNA using antibodies for ENG and GAPDH. The signal intensity ratios between the described proteins are indicated; *n* = 5. (C) ENG‐positive intensity on the indicated cells gauged by flow cytometry using anti‐ENG antibody; *n* = 4. A significant difference (*P* < 0.05) compared to exp‐CAF2‐shGFP cells is indicated by an asterisk. N.S.: not significant (D) Immunoblotting of the indicated cells expressing the described shRNA using antibodies for α‐SMA, pSmad2, Smad2/3, and α‐tubulin (α‐tub). The signal intensity ratios between the described proteins are indicated. (E) Real‐time PCR analysis of the indicated cells expressing the described shRNA using a primer specific for *TGFB1* gene. A significant difference (*P* < 0.05) compared to exp‐CAF2‐shGFP cells is indicated by an asterisk; *n* = 3, each performed in triplicate. (F) Immunoblotting of the indicated TGF‐β1‐treated (0.1 ng·mL^−1^ for 1 h) or ‐untreated cells expressing the described shRNA using antibodies for pSmad2, Smad2/3, ENG, and α‐tub; *n* = 4. (G) Relative pSmad2/Smad2/3 ratios of (F) are indicated. A significant difference (*P* < 0.05) compared to TGF‐β1‐treated exp‐CAF2‐shGFP cells is indicated by an asterisk; *n* = 4. (H) Immunoblotting of the indicated cells expressing the described shRNA using antibodies for pSmad1/5, Smad1, and α‐tub. The signal intensity ratios between the described proteins are indicated. Student's *t*‐test (A, C, E, G); error bars, SE (A, C, E, G).

Since activation of TGF‐β signaling augments TGF‐β production, generating a self‐stimulating autocrine signaling loop in myCAFs during tumor progression [19], we reasoned that ENG expression may be required for maintenance of TGF‐β autocrine signaling in these cells. To examine this possibility, we constructed two different lentiviral shRNA vectors (shENG‐1 and shENG‐2), which suppressed ENG expression in exp‐CAF2 cells at mRNA and protein levels by 50–54% and 73–84%, respectively, compared to the effect of the control GFP‐shRNA (shGFP; Fig. 2A,B). The proportion of ENG‐positive cells was also attenuated by the introduction of both shENG‐1 and ‐2 into exp‐CAF2 cells by 42–74%, as gauged by flow cytometry (Fig. 2C).

As anticipated, the ratio of phosphorylated Smad2 (pSmad2) to total Smad2/3 protein (pSmad2/Smad2/3), indicative of activated canonical TGF‐β signaling, and α‐SMA protein expression were decreased by 87–95% and 40–61%, respectively, in exp‐CAF2 cells expressing shENG‐1 or ‐2 (exp‐CAF2‐shENG‐1 or ‐shENG‐2 cells) relative to the control GFP‐shRNA (Fig. 2D). mRNA expression levels of *TGFB1* and *actin alpha 2, smooth muscle (ACTA2)* encoding α‐SMA were also attenuated in exp‐CAF2‐shENG cells by 38–60% and 85%, respectively (Fig. 2E and Fig. S2A). These findings indicate that ENG expression is required for the maintenance of TGF‐β‐Smad2/3 autocrine signaling, TGF‐β1 production, and the myofibroblastic state of myCAFs.

Next, we sought to elucidate whether the attenuated Smad2/3 signaling in exp‐CAF2‐shENG cells was attributable to the affected association of ENG with a TGF‐β ligand–receptor complex or the secondary effect of decreased levels of TGF‐β1 acting in an autocrine fashion. To test these possibilities, recombinant TGF‐β1 (0.1 ng·mL^−1^) was added to exp‐CAF2‐shENG cells for 1 h prior to measurement of phosphorylated Smad2. We observed a reduction of pSmad2/Smad2/3 ratio in these cells of 32–47%, as compared to that in exp‐CAF2‐shGFP cells treated with TGF‐β1 (Fig. 2F,G), indicating insufficient TGF‐β‐Smad2/3 signal transduction in TGF‐β1‐treated exp‐CAF2‐shENG cells. These findings are consistent with previous reports [22, 52, 53], indicating that the association of ENG with a TGF‐β1‐receptor complex mediates Smad2/3 signaling activation. In contrast, the pSmad2/Smad2/3 ratio increased in TGF‐β1‐treated exp‐CAF2‐shENG cells compared to the non‐TGF‐β1‐treated group (Fig. 2F,G). These observations suggest that, upon ligand stimulation, TGF‐β‐Smad2/3 signal could be initiated in exp‐CAF2‐shENG cells, although the relative capacity to transduce the signal is substantially limited in these cells.

Taken together, these findings indicate that ENG is required for efficient TGF‐β‐Smad2/3 signal transduction, presumably via association with a TGF‐β1‐receptor complex in myCAFs. Smad2/3 signaling would be further attenuated in exp‐CAF2‐shENG cells (expressing ENG protein by 16–27%) due to secondarily decreased production of TGF‐β1 that can stimulate Smad2/3 signaling in an autocrine fashion.

Previous studies demonstrated that ENG mediates proliferation and migration of ECs through BMP‐Smad1/5 signaling [24, 54]. Thus, we sought to determine whether the Smad1/5 pathway also serves as the downstream signaling for ENG in CAFs. We found that Smad1/5 protein phosphorylation was not attenuated but rather tended to be upregulated in exp‐CAF2‐shENG cells as compared to exp‐CAF2‐shGFP cells (Fig. 2H). Taken together with earlier findings, these data demonstrate that ENG‐mediated TGF‐β‐Smad2/3, but not ‐Smad1/5, signaling activation is required for TGF‐β1 production and the myofibroblastic trait in human breast myCAFs.

### 3.4 ENG expression is not affected by activation of TGF‐β signaling in CAFs

Since ENG expression is required for activation of TGF‐β signaling in CAFs, we aimed to determine whether ENG expression is induced by TGF‐β signaling. To address this, a construct encoding constitutively active TGF‐β1 [36], which can strongly upregulate *TGFB1* mRNA expression (Fig. S3A), was introduced into human mammary fibroblasts prior to measurement of *ENG* mRNA expression. We did not observe that *ENG* expression was upregulated, but was rather suppressed, in these fibroblasts (Fig. S3B). Treatment with recombinant TGF‐β1 also did not show any induction of ENG protein expression in human mammary fibroblasts (Fig. S3C), indicating that TGF‐β signaling activation is not sufficient to induce ENG expression in human mammary fibroblasts.

Next, we investigated whether TGF‐β‐Smad2/3 signaling is required for the maintenance of ENG expression in exp‐CAF2 cells. To address this, exp‐CAF2 cells were treated with shRNAs, one which inhibits the expression of *SMAD4*, a mediator of canonical TGF‐β‐Smad2/3 signaling (Fig. S3D), and the other, SB431542, an ALK5 inhibitor that suppresses Smad2 phosphorylation (Fig. S3E). Inhibition of TGF‐β‐Smad2/3 signaling by either of these treatments did not attenuate *ENG* mRNA expression in exp‐CAF2 cells (Fig. S3F,G), indicating that TGF‐β‐Smad2/3 signaling was not involved in ENG expression in these fibroblasts. Taken together, these findings demonstrate that the TGF‐β‐Smad2/3 signaling pathway is not required for the induction and maintenance of ENG expression in myCAFs.

### 3.5 Forced ENG expression facilitates TGF‐β‐Smad2/3 signaling in stromal fibroblasts upon TGF‐β1 treatment

We examined whether forced ENG expression promotes the conversion of human mammary fibroblasts into myCAFs. To this end, a construct encoding the human *ENG* cDNA was introduced into human mammary fibroblasts, resulting in significantly increased *ENG* mRNA (Fig. S4A). However, *TGFB1* and *ACTA2* mRNA expression levels, as well as Smad2 phosphorylation, were not upregulated in the resulting ENG‐expressing mammary fibroblasts relative to those in the control GFP‐expressing cells (Fig. S4B–D). These data indicate that TGF‐β‐Smad2/3 signaling and the myofibroblastic state are not induced by forced ENG expression in stromal fibroblasts.

Next, we investigated whether the forced ENG expression further enhances TGF‐β‐Smad2/3 signaling in TGF‐β1‐treated mammary fibroblasts. We found that TGF‐β1 treatment increased the pSmad2/Smad2/3 ratio by 1.5‐fold in ENG‐expressing mammary fibroblasts compared to those expressing control GFP (Fig. S4D). However, *TGFB1* and *ACTA2* mRNA expressions were not upregulated in the TGF‐β1‐treated ENG‐expressing mammary fibroblasts (Fig. S4B,C). These findings demonstrate that forced ENG expression augments TGF‐β‐Smad2/3 signaling transduction, but not myofibroblastic state in human mammary fibroblasts upon TGF‐β1 treatment.

### 3.6 ENG expression mediates the tumor‐ and metastasis‐promoting traits of CAFs

**Fig. 3:**
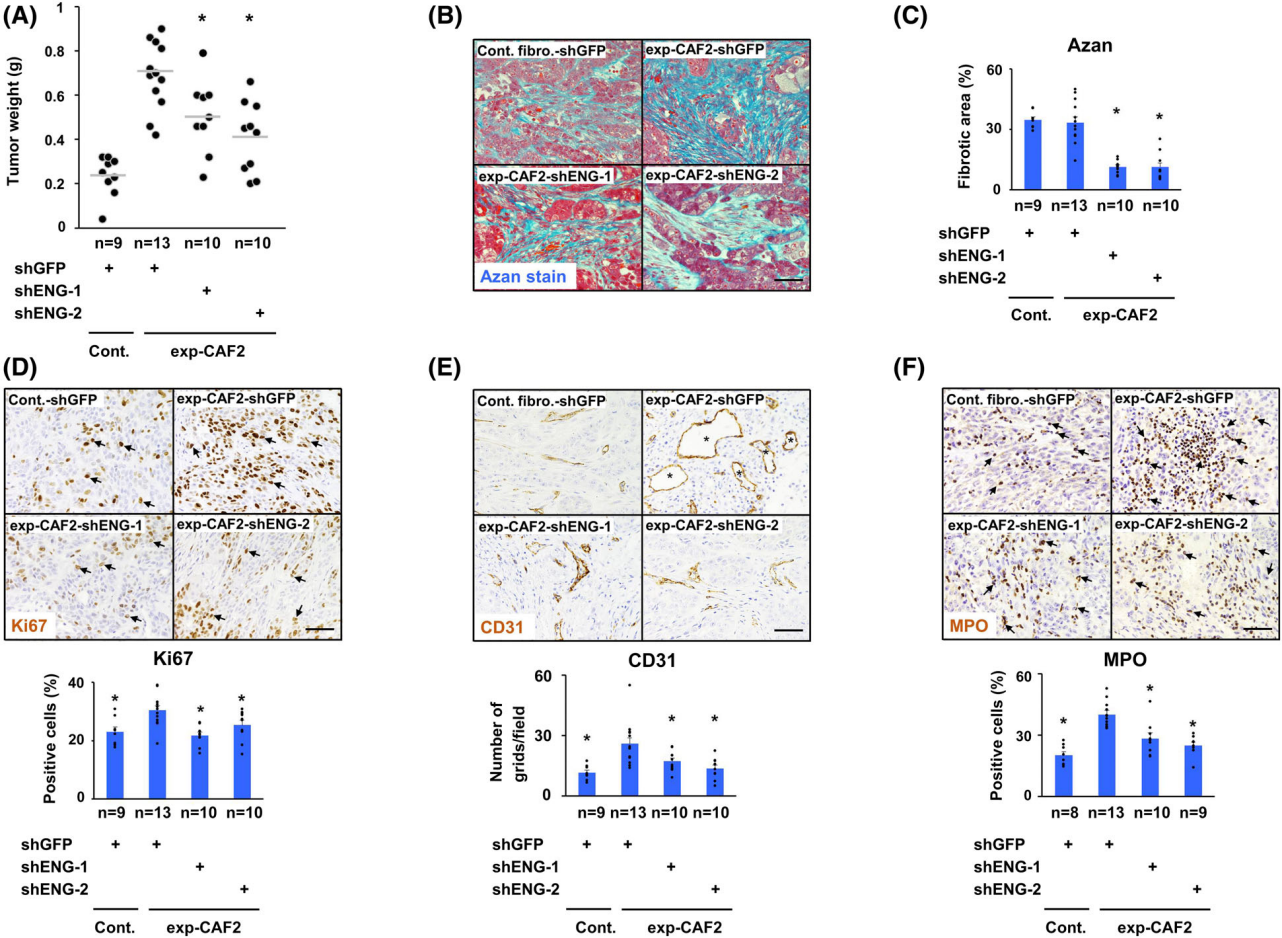
ENG expression mediates the tumor‐promoting trait in exp‐CAF2 cells. (A) Measurement of tumor weight at 21–25 days after subcutaneous co‐injection of ductal carcinoma *in situ* (DCIS) breast cancer cells and the indicated fibroblasts expressing the described shRNA into recipient NOD/Shi‐scid/IL‐2Rγnull (NOG) mice. Gray horizontal line means the average of all data points. A significant difference (*P* < 0.05) compared to experimentally generated CAF2 (exp‐CAF2)‐shGFP cells is indicated by an asterisk. Cont.: control fibroblasts (B–F) Sections prepared from DCIS tumors admixed with the indicated fibroblasts expressing the described shRNA were stained by Azan‐Mallory (B, C), anti‐Ki67 (D), ‐CD31 (E) or ‐myeloperoxidase (MPO) (F) antibodies. Ki67‐positive cancer cells (D) and MPO‐positive neutrophils (F) are depicted by triangular arrows. CD31‐positive capillary vessels (E) are indicated by asterisks. Azan‐positive fibrotic areas (%), the Ki67‐positive tumor cell proportions (%), the number of graticular points that overlapped with CD31‐positive capillary vessels, representing vascular lumen volume, and the MPO‐positive neutrophil proportions (%) were also quantified at ×400 magnification per field in a total of 10 fields per tumor section. A significant difference (*P* < 0.05) compared to the exp‐CAF2‐shGFP cell group is indicated by an asterisk. Mann–Whitney *U* test (A), Student's *t*‐test (C–F); error bars, SE (C–F); Scale bar, 50 μm (B, D, E, F).

Given that TGF‐β signaling activation is mediated by ENG expression in CAFs, we reasoned that ENG expression might be involved in the tumor‐promoting ability in these cells. To examine this possibility, human breast carcinoma DCIS cells [42] were co‐injected with exp‐CAF2 cells expressing each of the different shRNAs subcutaneously into immunodeficient NOG mice. We observed that the weights of tumors admixed with exp‐CAF2‐shENG‐1 or ‐shENG‐2 cells were significantly attenuated relative to those of the control exp‐CAF2‐shGFP cells (Fig. 3A).

To address how the inhibited *ENG* expression in CAFs contributed to the attenuated mammary carcinoma growth, we prepared sections from different tumors and performed Azan‐Mallory staining for collagen fibers. Desmoplastic areas indicated as blue were significantly reduced in sections of tumors containing exp‐CAF2‐shENG‐1 or ‐2 cells (Fig. 3B,C). Tumor sections were also stained with antibodies for Ki67, cleaved poly‐ADP‐ribose polymerase (cPARP), CD31, and myeloperoxidase (MPO) to evaluate tumor cell proliferation, apoptosis, angiogenesis, and inflammation, respectively. We observed significant decreases in Ki67‐positive tumor cell proportions, CD31‐positive blood vessel number and volume, and MPO‐positive neutrophil proportions in tumors admixed with exp‐CAF2‐shENG‐1 or ‐2 cells (Fig. 3D–F and Fig. S5A). On the other hand, the number of cancer cells positive for cPARP, an apoptosis marker, was comparable among the groups (Fig. S5B). Collectively, these data indicate that ENG expression is required for CAFs to promote tumor cell proliferation, angiogenesis, neutrophil infiltration, and ECM remodeling in tumors.

**Fig. 4:**
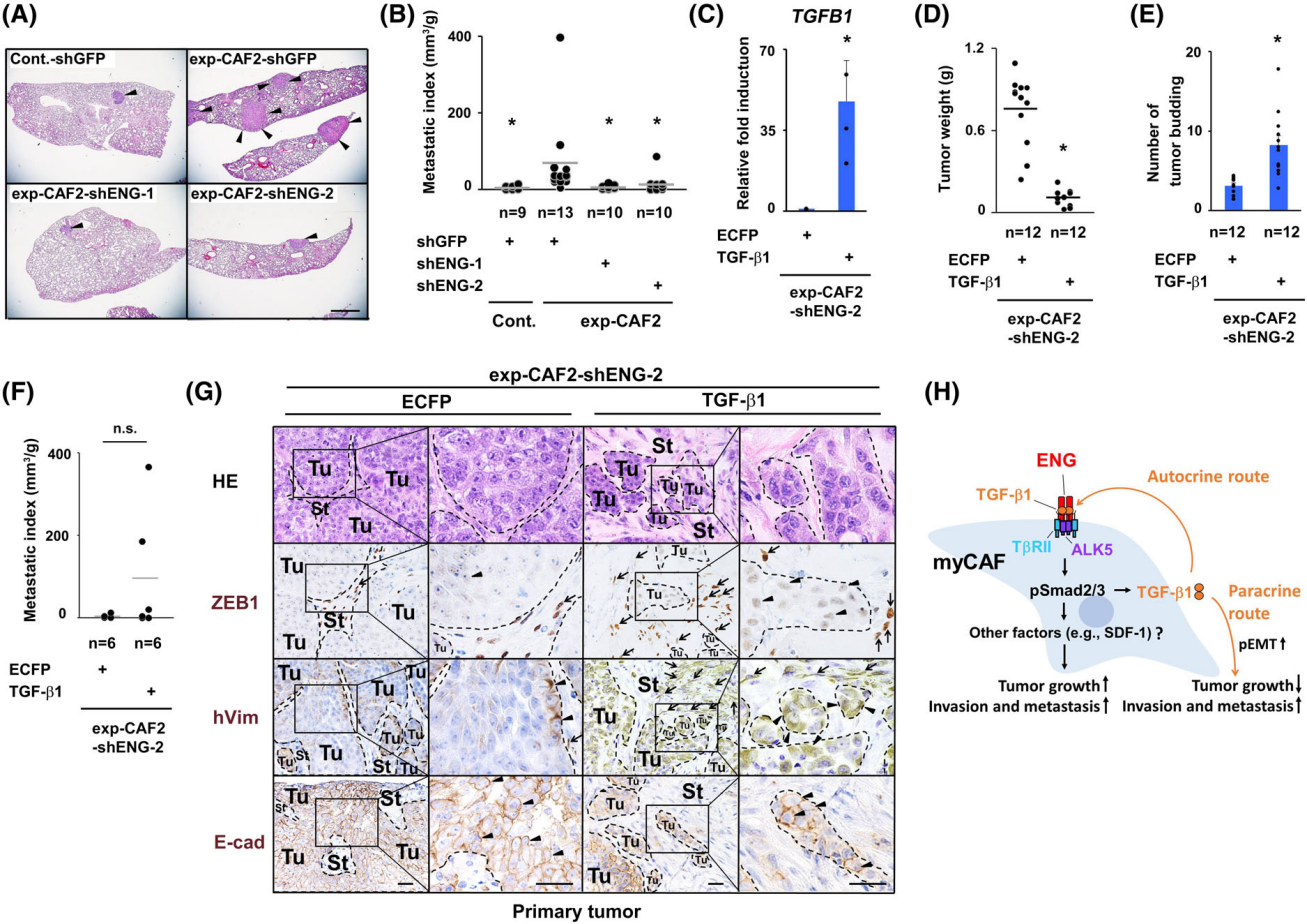
ENG expression mediates the metastasis‐promoting trait in exp‐CAF2 cells. (A) HE staining of the lungs with metastatic nodules (arrowheads). Ductal carcinoma *in situ* (DCIS) breast cancer cells and the indicated fibroblasts expressing the described shRNA were injected subcutaneously into NOD/Shi‐scid/IL‐2Rγnull (NOG) mice. The lungs were dissected from these mice 65 days after injection. Scale bar, 1 mm. Cont.‐shGFP: *n* = 10, exp‐CAF2‐shGFP: *n* = 13, exp‐CAF2‐shENG1: *n* = 10, exp‐CAF2‐shENG2: *n* = 10. Cont.: control fibroblasts, exp‐CAF2: experimentally generated CAF2, ENG: endoglin (B) The metastatic index was employed as the ratio of metastasis formation, gauged by pulmonary nodule volume (mm^3^) in the lungs, relative to the weight (g) of the primary tumors. Gray horizontal line means the average of all data points. A significant difference (*P* < 0.05) compared to exp‐CAF2‐shGFP cells is indicated by an asterisk. (C) Real‐time PCR analysis of exp‐CAF2‐shENG‐2 cells expressing a constitutively active *TGFB1* cDNA construct using *TGFB1* gene‐specific primers. A significant difference (*P* < 0.05) compared to exp‐CAF2‐shENG‐2 cells expressing the control *ECFP* gene is indicated by an asterisk; *n* = 3. (D) Attenuated growth of primary tumors containing active TGF‐β1‐expressing exp‐CAF2‐shENG‐2 cells. ECFP‐ or active TGF‐β1‐expressing exp‐CAF2‐shENG‐2 cells were comingled with DCIS cells prior to subcutaneous injection into NOG mice. Primary tumors were dissected from these mice 30 days after injection. The weight (g) of primary tumors was measured. Gray horizontal line means the average of all data points. A significant difference (*P* < 0.05) calculated using the Mann–Whitney U test, compared to exp‐CAF2‐shENG‐2 cells expressing the control ECFP gene, is indicated by an asterisk. (E, F) Increased tumor budding (E) and metastatic index (F) in tumors admixed with exp‐CAF2‐shENG‐2 cells expressing an active *TGFB1* construct. ECFP‐ or active TGF‐β1‐expressing exp‐CAF2‐shENG‐2 cells were comingled with DCIS cells prior to subcutaneous injection into NOG mice. 30‐day‐old primary tumors were dissected from these mice for HE staining for measurement of tumor budding, followed by dissection of the lungs at 50–53 days after injection. A significant difference (*P* < 0.05) compared to exp‐CAF2‐shENG‐2 cells expressing the control *ECFP* gene is indicated by an asterisk (E). The volume (mm^3^) of metastatic nodules generated in the lungs was calculated, and the metastatic index was employed. Gray horizontal line means the average of all data points (F). N.S.: not significant (G) HE staining and immunohistochemistry of sections of the above 30‐day‐old primary tumors using antibodies for ZEB1, human vimentin (hVim) and E‐cadherin (E‐cad). ZEB1‐, hVim‐ or E‐cad‐positive cancer cells are marked by arrowheads. ZEB1‐ or hVim‐positive fibroblasts are indicated by simple arrows. Tu, tumor area; St, stroma area; Scale bar, 30 μm. *n* = 6 for each group. (H) Schematic representation of ENG‐primed TGF‐β autocrine and paracrine signaling in human breast myCAFs through TGF‐β1 production. Increased ENG expression in human breast myCAFs is required for tumor‐ and metastasis‐promoting ability in these fibroblasts. ENG facilitates TGF‐β‐Smad2/3 signaling in myCAFs, presumably through association with a TGF‐β ligand–receptor complex, leading to self‐stimulating TGF‐β1 production. Stromal TGF‐β1, in turn, acts on nearby carcinoma cells in a paracrine manner to induce partial epithelial‐mesenchymal transition (pEMT), resulting in suppression of primary tumor growth and, conversely, promotion of invasion and metastasis. Other factors (e.g., SDF‐1) induced by ENG‐primed TGF‐β autocrine signaling are also likely to mediate primary tumor growth promotion by myCAFs. Mann–Whitney *U* test (B, D, F), Student's *t*‐test (C, E); error bars, SE (C, E).

As CAFs have the ability to promote invasion and metastasis of human breast cancer cells [21], we sought to determine whether ENG expression on CAFs contributes to their invasion‐ and metastasis‐promoting ability. To address this, we resected 21–25‐day‐old DCIS tumors admixed with exp‐CAF2‐shGFP, exp‐CAF2‐shENG‐1, or ‐2 cells grown subcutaneously in recipient mice, followed by the use of lung tissues to evaluate metastatic nodules at 65 days after injection. The metastatic index, indicative of metastatic nodule volume (mm^3^) in the lungs relative to primary tumor weight (g), was also calculated to consider the secondary effects associated with the attenuated primary tumor growth. Lung metastatic nodules and the metastatic index were significantly decreased in mice bearing tumors admixed with exp‐CAF2‐shENG‐1 or ‐2 cells relative to those with exp‐CAF2‐shGFP cells (Fig. 4A,B). Taken together, these findings indicate ENG expressed on myCAFs is required for their tumor‐ and metastasis‐promoting ability.

### 3.7 TGF‐β1 produced by ENG‐primed TGF‐β‐Smad2/3 autocrine signaling in myCAFs induces pEMT on apposed carcinoma cells in a paracrine manner

Given the decreased *TGFB1* expression in exp‐CAF2‐shENG cells (Fig. 2E), we sought to determine whether the attenuated tumor‐ and metastasis‐promoting ability in these fibroblasts is due to the decreased TGF‐β1 levels. To address this, we introduced a constitutively active *TGFB1* construct into these cells, resulting in a 47.6‐fold upregulation of *TGFB1* mRNA expression (Fig. 4C), prior to co‐injection with DCIS cells subcutaneously into recipient mice. Since Smad2/3 signal transduction capacity is substantially attenuated in exp‐CAF2‐shENG cells (Fig. 2F,G), TGF‐β1 released from these fibroblasts could not significantly influence the Smad2/3 pathway in an autocrine manner. Therefore, stromal TGF‐β1 more likely affects apposed cancer cells in a paracrine manner. Indeed, we observed much reduced weights of primary tumors admixed with exp‐CAF2‐shENG‐2 cells expressing TGF‐β1 relative to those with control ECFP (Fig. 4D), indicating that primary tumor growth was suppressed by paracrine stromal TGF‐β1 signaling. TGF‐β1‐suppressed tumor cell proliferation and growth has been consistently documented by a large number of previous reports [20].

In contrast, we observed a significant increase in tumor budding, indicative of a group of invading tumor cells and an increasing trend in lung metastasis in mice bearing tumors containing TGF‐β1‐expressing exp‐CAF2‐shENG‐2 cells (Fig. 4E,F). The injected exp‐CAF2 cells (human vimentin‐positive) were detected in considerable numbers in 30‐day‐old primary tumors (Fig. 4G), demonstrating that exp‐CAF2 cells present in advanced tumors could continuously produce TGF‐β1 to influence nearby carcinoma cells. Increased ZEB1‐ and vimentin‐positive tumor cells with decreased membrane E‐cadherin staining were also present in these tumors, indicating pEMT to be induced in tumor cells by stromal TGF‐β1 (Fig. 4G).

Taken together, these findings indicate that myCAF‐producing TGF‐β1 induces pEMT of apposed cancer cells in a paracrine fashion, resulting in the suppression of primary tumor growth and, conversely, promotion of invasion and lung metastasis formation. Therefore, the decreased TGF‐β1 production in exp‐CAF2‐shENG cells contributes to attenuated lung metastasis, but not to decreased primary tumor growth. exp‐CAF2‐shENG cells also showed attenuated mRNA expression levels of α and β alternative splicing forms of *CXCL12/stromal cell‐derived factor‐1* (*SDF‐1*; Fig. S5C), which could mediate CAF‐promoted primary tumor growth based on our previous studies [21, 41]. This finding thus suggests that other factors involving SDF‐1 produced by ENG‐mediated TGF‐β autocrine signaling presumably mediate primary tumor growth promotion via myCAFs (Fig. 4H).

## 4 Discussion

Activation of canonical TGF‐β signaling is crucial for the induction and maintenance of myofibroblasts, the major hallmark of activated fibroblasts in wound healing, fibrosis, and tumorigenesis [13, 14, 20].

We previously reported that the myofibroblastic trait is stably maintained in human breast CAFs due to the establishment of TGF‐β‐Smad2/3 autocrine signaling without ongoing interactions with cancer cells in culture [19]. However, the mechanism(s) by which such autocrine TGF‐β signaling and the myofibroblastic trait are maintained in myCAFs remain poorly understood. ENG, a TGF‐β superfamily coreceptor, is essential for smooth muscle cell specification, contributing to vascular integrity, and its expression is often upregulated in myofibroblasts in settings of tissue injury, fibrosis, and cancer [28, 30, 34]. Thus, we investigated the roles of ENG in TGF‐β autocrine signaling in myCAFs.

Recent studies have demonstrated that stromal ENG expression in tumors is associated with poorer outcomes in breast and colon cancer patients [25, 32], as well as mediating resistance to androgen depletion therapy in prostate cancer patients [33]. However, whether ENG expression mediates TGF‐β signaling activation and tumor‐promoting abilities in myCAFs remained unclear. In this study, we demonstrated that stronger ENG staining in myCAFs in tumor stroma is associated with a poorer outcome in breast cancer patients, and that ENG expression is progressively upregulated on human breast CAFs during tumor progression. We also found previously unrecognized roles of ENG required for primary tumor‐ and metastasis‐promoting traits of myCAFs, at least via producing TGF‐β1 that acts through autocrine and paracrine mechanisms (Fig. 4H).

We observed that *ENG* suppression by shRNA in myCAFs not only attenuated TGF‐β‐Smad2/3 signaling and TGF‐β1 production but also inhibited the ability of these fibroblasts to promote tumor growth when co‐injected with human breast cancer cells subcutaneously into recipient mice. This attenuated tumor growth was due to decreased tumor cell proliferation, neoangiogenesis, neutrophil infiltration, and ECM remodeling (Fig. 3A–F).

Inhibition of *ENG* expression in CAFs also attenuated their ability to promote lung metastasis spontaneously formed by breast cancer cells (Fig. 4A,B). ENG facilitates TGF‐β‐Smad2/3 signaling, presumably via association with a TGF‐β ligand–receptor complex in myCAFs, resulting in the production of self‐stimulating TGF‐β1 in an autocrine manner. Given the significantly attenuated *TGFB1* expression levels in exp‐CAF2‐shENG cells, we speculated that the decreased TGF‐β1 production contributed to the suppression of primary tumor growth and metastasis by ENG reduction in CAFs. To test this, an active *TGFB1* construct was introduced in exp‐CAF2‐shENG cells with limited Smad2/3 signaling transduction capacity, resulting in an inability of the TGF‐β1 product to influence the Smad2/3 signal pathway in these cells in a self‐stimulating autocrine fashion. Indeed, we observed a decrease in primary tumor growth and, conversely, an increased collective invasion of E‐cadherin‐, ZEB1‐, and vimentin‐positive tumor budding and metastasis to the lungs. These data indicate that paracrine stromal TGF‐β1 signaling suppresses primary tumor growth but promotes tumor invasion and metastasis via pEMT induction, demonstrating that decreased TGF‐β1 production in exp‐CAF2‐shENG cells, at least, contributes to an attenuation of tumor invasion and metastasis. Other factors (e.g., SDF‐1) presumably induced by ENG‐primed TGF‐β autocrine signaling would also mediate the primary tumor growth promoted by myCAFs (Fig. 4H).

Our previous work indicated that TGF‐β‐Smad2/3 and SDF‐1‐CXCR4 autocrine signaling converges in a self‐stimulating and cross‐communicating fashion in exp‐CAF2 cells [19]. TGF‐β‐Smad2/3 signaling activation thus induces and is required for the maintenance of increased SDF‐1 expression in these fibroblasts. CAF‐produced SDF‐1, in turn, plays crucial roles in boosting tumor growth and metastasis [21, 41]. Indeed, we observed significantly attenuated *SDF‐1* expression in exp‐CAF2‐shENG cells (Fig. S5C). This decreased stromal SDF‐1 production may, therefore, mediate the decreased growth of primary tumors admixed with exp‐CAF2‐shENG cells. SDF‐1 induced by ENG‐primed TGF‐β autocrine signaling in CAFs might also contribute to their metastasis‐promoting effect.

myCAFs have the ability to either promote or suppress tumor growth [55, 56, 57]. TGF‐β signaling activation in myCAFs mediates the acceleration of colon tumor growth and metastasis [18]. TGF‐β‐activated epidermal growth factor receptor signaling in myCAFs contributes to the promotion of tumor growth and metastasis of pancreatic ductal adenocarcinoma (PDAC) [58]. Finally, TGF‐β‐dependent leucine‐rich‐repeat‐containing protein 15‐positive myCAFs also play roles in promoting tumor growth by impeding tumor immunity [59].

On the other hand, several reports have suggested the tumor‐suppressive ability of myCAFs. Depletion of α‐SMA myofibroblasts in an autochthonous PDAC mouse model resulted in increased vascularity, reduced immune infiltration, and decreased survival [60]. Deletion of the type 1 collagen gene in myCAFs accelerated tumor progression and worsened survival by altering tumor immunity in a PDAC murine model [61]. These context‐dependent roles of myCAFs may be due to heterogeneities in myCAF populations, presumably influenced by their heterogeneous cellular origins and the diversity in interactions with various carcinoma cells with distinct epi/genetic alterations and transcriptomes.

We show that ENG expression is required for the maintenance of TGF‐β‐Smad2/3, but not TGF‐β‐Smad1/5, signaling in breast CAFs. A previous study also demonstrated that ENG is required for induction of Smad2/3 phosphorylation in TGF‐β1‐treated human umbilical vein ECs [49]. In contrast, ENG reportedly mediates BMP9‐induced Smad1 signaling in human colonic and pancreatic CAFs, as well as in ECs [25, 62]. The absence of Smad1/5 signaling activation mediated by ENG in our breast CAFs would be due to undetectable endogenous *BMP9* and *BMP10* expression and the context‐dependent ENG interactions with ALK5 and ALK1 upon TGF‐β stimulation.

A recent study demonstrated that treatment with a TRC105 antibody, which inhibits the interaction of BMP9 with ENG, on CAFs could not suppress tumor growth in a syngeneic murine model of pancreatic cancer [62], indicating ENG‐mediated BMP–Smad1/5 signaling has little tumor‐promoting ability. The authors also indicated that deletion of the *ENG* gene specifically in collagen type 1α1‐expressing stromal cells resembling CAFs produced very little inhibition of the growth of murine pancreatic cancer cells injected orthotopically into mice [62]. myCAFs are composed of various stromal fibroblast populations, and collagen type 1α1 is expressed in some myCAFs. The tumor‐promoting roles of ENG on myCAFs not expressing collagen type 1α1, therefore, remain to be determined.

We observed that *ENG* expression was not induced in CAFs through activation of TGF‐β signaling (Fig. S3B). This finding demonstrates that increased *ENG* expression, independently regulated by TGF‐β signaling activation, mediates activation of TGF‐β signaling in CAFs. Elucidation of the molecular mechanisms underlying induction of *ENG* expression in CAFs awaits future studies.

### 4.1 Limitations of the study

Although we observed that increased ENG expression in myCAFs mediates their tumor‐ and metastasis‐promoting ability, precisely how ENG expression is upregulated in these fibroblasts remains an unresolved issue. We demonstrated that TGF‐β1 produced by ENG‐primed TGF‐β‐Smad2/3 autocrine signaling in myCAFs induced pEMT in apposed carcinoma cells in a paracrine fashion, resulting in the promotion of invasion and metastasis. However, which factors produced by such ENG‐primed TGF‐β autocrine signaling contributed to the primary tumor growth promotion by myCAFs remain to be elucidated in future studies. This study also failed to determine whether the cell‐surface ENG expression marks CAFs with greater tumor‐ and metastasis‐promoting ability, leading to the characterization of bona fide tumor‐promoting fibroblasts within myCAFs, presumably composed of various fibroblast subpopulations with distinct functions.

## 5 Conclusions

Our findings indicate that abundant ENG on myCAFs is associated with poorer outcomes for breast cancer patients, and ENG expression in fibroblasts is required for their primary tumor‐ and metastasis‐promoting ability. ENG‐primed TGF‐β‐Smad2/3 signaling in myCAFs facilitates the production of self‐stimulating TGF‐β1 in an autocrine manner. The TGF‐β1 product also acts on cancer cells through a paracrine mechanism to induce pEMT, resulting in suppression of tumor cell proliferation and, conversely, in stimulation of invasion and metastasis. Other factors presumably generated by the ENG‐primed TGF‐β‐Smad2/3 autocrine signaling in myCAFs would also mediate primary tumor growth promotion by these fibroblasts. Therefore, this increased ENG expression may have the potential to serve as a therapeutic target for tumor‐ and metastasis‐promoting myCAFs.

## Conflict of interest

The authors declare no conflict of interest.

## Author contributions

AO mainly conceived and designed the project. AT, TYo, TYa, and YD acquired the data of Fig. 1D and Fig. S1D. SO, YM, ZW, AA, YI, and TS acquired the rest of all data. SO, YM, TS, and AO analyzed and interpreted the data and wrote the paper.

## Peer review

The peer review history for this article is available at https://www.webofscience.com/api/gateway/wos/peer‐review/10.1002/1878‐0261.70074.

## Supporting information


**Fig. S1.** Endoglin staining in human mammary tumor stroma.

**Fig. S2.** *Actin alpha 2* expression is attenuated in exp‐CAF2‐shENG cells.

**Fig. S3.** Endoglin expression is not induced and maintained via TGF‐β‐Smad2/3 signaling in carcinoma‐associated fibroblasts.

**Fig. S4.** Forced endoglin expression further facilitates TGF‐β‐Smad2/3 signaling in human mammary fibroblasts upon TGF‐β1 treatment.

**Fig. S5.** Various changes in tumors including exp‐CAF2‐shENG cells.

**Table S1.** A list of antibodies, shRNA target sequences and PCR primers.
**Table S2.** Association of stromal endoglin staining with clinical parameters in breast cancer patients.
**Table S3.** Multivariable analysis of stromal endoglin staining and other prognostic factors in breast cancer patients.
**Table S4.** Prognostic value of endoglin staining in breast cancer patients by a regression model.

## Data Availability

Data sharing is not applicable to this article as no new data were created or analyzed in this study.
