# Supplementary material for: Endoglin mediates the tumor‐ and metastasis‐promoting traits of stromal myofibroblasts in human breast carcinomas
Source: Mol Oncol. 2025 Jul 11;19(9):2557–73. doi: 10.1002/1878-0261.70074 (PMC12420333; doi:10.1002/1878-0261.70074)
Supplement: Supplementary file 6 — Table S1. A list of antibodies, shRNA target sequences and PCR primers. Table S2. Association of stromal endoglin staining with clinical parameters in breast cancer patients. Table S3. Multivariable analysis of stromal endoglin staining and other prognostic factors in breast cancer patients. Table S4. Prognostic value of endoglin staining in breast cancer patients by a regression model. [file MOL2-19-2557-s001.docx]

**Supporting Information:**

**Table S1 A list of antibodies, shRNA target sequences and PCR primers**

**Table S2** **Association of stromal ENG staining with clinical parameters in breast cancer patients**

**Table S3 Multivariable analysis of stromal ENG staining and other prognostic factors in breast cancer patients**

**Table S4 Prognostic value of ENG staining in breast cancer patients by a regression model**

**Fig. S1 ENG staining in human mammary tumor stroma.**

**Fig. S2 ACTA2 expression is attenuated in** **exp-CAF2-shENG cells.**

**Fig. S3 ENG expression is not induced and maintained via TGF-β-Smad2/3 signaling in CAFs.**

**Fig. S4 Forced ENG expression further facilitates TGF-β-Smad2/3 signaling in human mammary fibroblasts upon TGF-β1 treatment.**

**Fig. S5 Various changes in tumors including exp-CAF2-shENG cells.**

**Sup. Table 1**

|  | | | | | |
| --- | --- | --- | --- | --- | --- |
|  |  | **Total** | **Strongly/weakly positive stromal ENG staining** | **Negative stromal ENG staining** | ***P*-value**  **(Strongly/weakly positive vs. negative staining)** |
|  |  | **n = 232** | **n = 67** | **n = 165** |  |
| **Age** | |  |  |  |  |
|  | **< 65** | **175** | **49** | **126** | **0.6165** |
|  | **65 =<** | **57** | **18** | **39** |  |
| **Grading** | |  |  |  |  |
|  | **0** | **40** | **10** | **30** | **> 0.9999** |
|  | **1** | **76** | **24** | **52** |  |
|  | **2** | **60** | **17** | **43** |  |
|  | **3** | **56** | **16** | **40** |  |
| **pT factor** | |  |  |  |  |
|  | **T1** | **82** | **24** | **58** | **> 0.9999** |
|  | **T2-T3** | **150** | **43** | **107** |  |
| **pN factor** | |  |  |  |  |
|  | **N0** | **132** | **33** | **99** | **0.1456** |
|  | **N1-N2** | **100** | **34** | **66** |  |
| **ER status** | |  |  |  |  |
|  | **Negative** | **63** | **22** | **41** | **0.2544** |
|  | **Positive** | **169** | **45** | **124** |  |
| **HER-2/neu status** | |  |  |  |  |
|  | **Negative** | **191** | **55** | **136** | **> 0.9999** |
|  | **Positive** | **41** | **12** | **29** |  |
|  | ****P* < 0.05 (Fisher's exact test)** | | | | |

**Sup. Table 2**

**
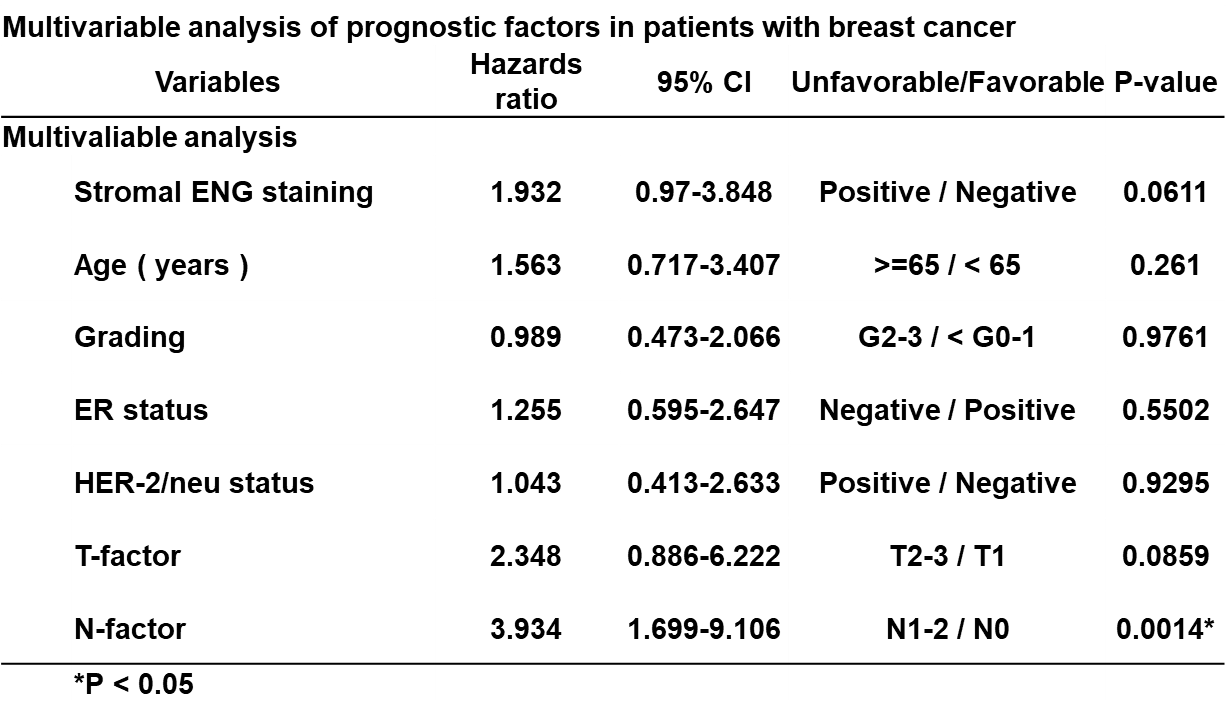
**

**Sup. Table 3**

**
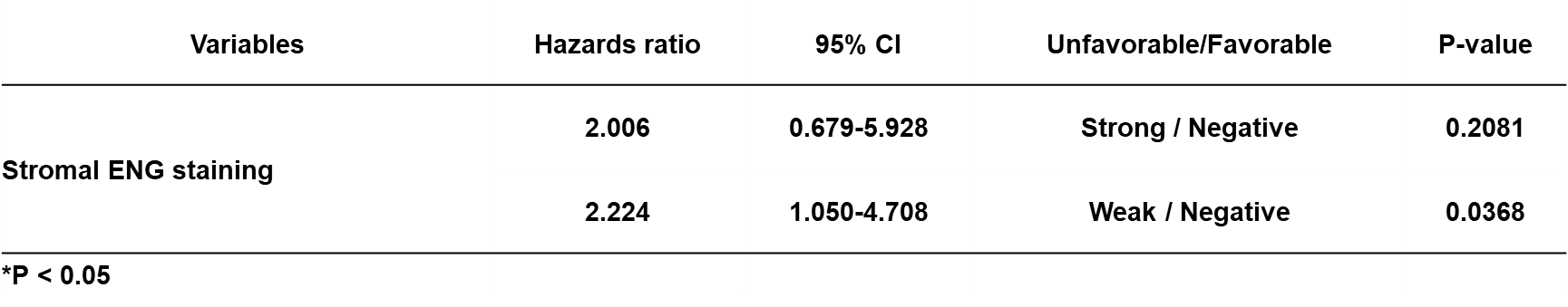
**

**
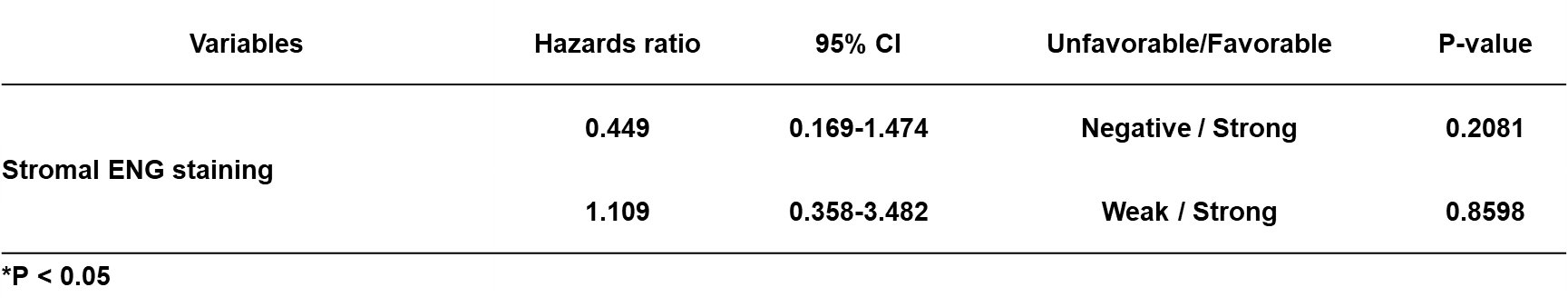
**

**Sup. Table 4**

**Antibodies**

| **Antibodies**  **(Clone name)** |  | **Source** | **Purpose** |
| --- | --- | --- | --- |
| Endoglin-APC (166707) | Mouse IgG1 | R&D systems (FAB10971A) | FCM |
| Endoglin (3A9) | Mouse IgG1 | Cell Signaling Technology (#14606) | WB  IHC (dilution: 1:400, antigen retrieval: pH6) |
| α-SMA (1A4) | Mouse IgG2a | Dako (M0851) | WB  IHC (dilution: 1:200, antigen retrieval: pH6) |
| Smad2/3 | Mouse IgG1, κ | BD Biosciences (610842) | WB |
| Phosphor-Smad2 (138D4) | Rabbit IgG | Cell Signaling Technology (#3108) | WB |
| Smad1 | Rabbit IgG | Cell Signaling #6944S | WB |
| Phosphor-Smad1/5 (Ser463/465) | Rabbit mAb | Cell Signaling Technology #9516S | WB |
| α-tubulin | Mouse IgG1 | SIGMA (T5168) | WB |
| α-tubulin (DM1A) | Mouse IgG | Abcam (ab7291) | WB |
| CD31 | Mouse IgG1 | Leica (NCL-CD31-1A10) | IHC (dilution: 1:100, antigen retrieval: pH6) |
| Periostin | Mouse IgG1κ | Adipogen  (AG-20B-0033-C100) | IHC (dilution: 1:200, antigen retrieval: pH6) |
| Ki67 | Mouse IgG | Dako (M7240) | IHC (dilution: 1:150, antigen retrieval: pH6) |
| cPARP | Rabbit IgG | Cell Signaling Technology (5625s) | IHC (dilution: 1:150, antigen retrieval: pH6) |
| MPO | Rabbit IgG polyclonal | Dako (A0398) | IHC (dilution: 1:400, antigen retrieval: pH6) |
| ZEB-1 | Rabbit IgG polyclonal | SIGMA (HPA027524) | IHC (dilution: 1:100, antigen retrieval: pH6) |
| Vimentin | Mous IgG1κ | Santa Cruz (sc-6260) | IHC (dilution: 1:100, antigen retrieval: pH6) |
| E-cadherin | Rabbit IgG | abcam (ab40772) | IHC (dilution: 1:100, antigen retrieval: pH6) |

FCM: flow cytometry, IHC: immunohistochemistry, WB: western blotting

**Antibodies used for double staining**

α-SMA/Endoglin

|  | α-SMA  (DAKO M0851) | Endoglin (3A9)  (Cell Signaling #14606) |
| --- | --- | --- |
| Antigen retrieval pH | 6 | 6 |
| Dilution | 1:200 | 1:400 |
| Blocking　antibodies | Normal Rabbit Serum  (DAKO X0902) | Normal Goat Serum for Immunochemistry  (WAKO 143-06561) |
| Secondary antibodies | Polyclonal Rabbit Anti-Mouse Immunoglobulins/AP  (DAKO D0314) | EnVision+ System- HRP Labelled Polymer Anti-mouse  (DAKO K4001) |

CD31/Endoglin

|  | CD31   (Leica NCL-CD31-1A10) | Endoglin (3A9)  (Cell Signaling #14606) |
| --- | --- | --- |
| Antigen retrieval pH | 6 | 6 |
| Dilution | 1:100 | 1:400 |
| Blocking　antibodies | Normal Rabbit Serum  (DAKO X0902) | Normal Goat Serum for Immunochemistry  (WAKO 143-06561) |
| Secondary antibodies | Polyclonal Rabbit Anti-Mouse Immunoglobulins/AP  (DAKO D0314) x30 | EnVision+ System- HRP Labelled Polymer Anti-mouse  (DAKO K4001) |

Periostin/Endoglin

|  | Periostin   (AdioiGen  AG-20B-0033-C100) | Endoglin (3A9)  (Cell Signaling #14606) |
| --- | --- | --- |
| Antigen retrieval pH | 6 | 6 |
| Dilution | 1:200 | 1:400 |
| Blocking　antibodies | Normal Rabbit Serum  (DAKO X0902) | Normal Goat Serum for Immunochemistry  (WAKO 143-06561) |
| Secondary antibodies | Polyclonal Rabbit Anti-Mouse Immunoglobulins/AP  (DAKO D0314) | EnVision+ System- HRP Labelled Polymer Anti-mouse  (DAKO K4001) |

**shRNA target sequences**

| **Sequence name** | **Sequence** | **Reference** |
| --- | --- | --- |
| GFP-shRNA | 5’-GCAAGCTGACCCTGAAGTTCA-3’ | (Orimo et al., 2005) |
| Smad4-shRNA1 | 5’-UAUCCAUC-  AACAGUAACAAUAGGGC -3’ | (Koinuma et al., 2009) |
| Smad4-shRNA2 | 5’-UUACAUUCCAACUGCACAC-  CUUUGC-3’ | (Hoshino et al., 2015) |
| Endoglin-shRNA1 | 5’-AACACCACAGAGCTGCCATCC-3’ | (Sanz-Rodriguez et al., 2004) |
| Endoglin-shRNA2 | 5’-ATGACCCTGGTACTAAAGAAA-3’ | (This paper) |

**PCR primers**

| **Gene Name** | **Forward/Reverse** |
| --- | --- |
| ACTA2 | 5’-GTGTGTGACAATGGCT-3’  /5’-TGGTGATGATGCCATG-3’ |
| Human TGFB1 | 5’-ACTGCAAGTGGACATCAACG-3’  /5’-TGCGGAAGTCAATGTA-3’ |
| Swine TGFB1 | 5’-ACTACGCCAAGGAGGTCACC-3’  /5’-AGCTCCACGTGCTGCTCCAC-3’ |
| TGFBR3 | 5’ACATGGATAAGAAGCGATTCAGC-3’  /5’-AACGCAATGCCCATCACGGTTAG-3’ |
| Endoglin | 5’-TTGTCTTGCGCAGTGCTTAC-3’  /5’-TGAGGCAGTGCACCTTTTTC-3’ |
| BMP9 | 5’-CCTGGGCACAACAAGGAC-3’  /5’-CCTTCCCTGGCAGTTGAG-3’ |
| BMP10 | 5’-CAGCAGTGGACGGCTAGAAA-3’  /5’-TCGGGCAGTGGAGTCATAGA-3’ |
| B2M | 5’-TGAGTGCTGTCTCCATGTTTGA-3’  /5’-TCTGCTCCCCACCTCTAAGTTG-3’ |
| GAPDH | 5’-ACCCAGAAGACTGTGGATGG-3’  /5’-TCTAGACGGCAGGTCAGGTC-3’ |

References

Hoshino, Y., Nishida, J., Katsuno, Y., Koinuma, D., Aoki, T., Kokudo, N., Miyazono, K., and Ehata, S. (2015). Smad4 Decreases the Population of Pancreatic Cancer-Initiating Cells through Transcriptional Repression of ALDH1A1. Am J Pathol *185*, 1457-1470.

Koinuma, D., Tsutsumi, S., Kamimura, N., Imamura, T., Aburatani, H., and Miyazono, K. (2009). Promoter-wide analysis of Smad4 binding sites in human epithelial cells. Cancer Sci *100*, 2133-2142.

Orimo, A., Gupta, P.B., Sgroi, D.C., Arenzana-Seisdedos, F., Delaunay, T., Naeem, R., Carey, V.J., Richardson, A.L., and Weinberg, R.A. (2005). Stromal fibroblasts present in invasive human breast carcinomas promote tumor growth and angiogenesis through elevated SDF-1/CXCL12 secretion. Cell *121*, 335-348.

Sanz-Rodriguez, F., Guerrero-Esteo, M., Botella, L.M., Banville, D., Vary, C.P., and Bernabeu, C. (2004). Endoglin regulates cytoskeletal organization through binding to ZRP-1, a member of the Lim family of proteins. J Biol Chem *279*, 32858-32868.
